# Effectiveness of Different Washing Strategies on Pesticide Residue Removal: The First Comparative Study on Leafy Vegetables

**DOI:** 10.3390/foods11182916

**Published:** 2022-09-19

**Authors:** So-Jin Yang, Sujin Mun, Hye Jin Kim, Sue Ji Han, Do Woo Kim, Bae-Sik Cho, Ae Gyeong Kim, Duck Woong Park

**Affiliations:** Health and Environment Research Institute of Gwangju, 584, Mujin-daero, Seo-gu, Gwangju 61954, Korea

**Keywords:** pesticide residue, leafy vegetable, ssamchoo, boiling, detergent

## Abstract

Leafy vegetables are used in various cuisines worldwide; however, as they cannot be peeled and their leaf surface area is large, the risk of retaining pesticide residues on these vegetables is relatively higher than on others. To our knowledge, this is the first comparative study to reveal the effect of removing pesticide residues from five artificially contaminated leafy vegetables (lettuce, perilla leaves, spinach, crown daisy, and ssamchoo (*Brassica lee* ssp. *namai*)) using different removal methods. The percent reduction range for each method was 43.7–77.0%, and the reduction range for the five leafy vegetables was 40.6–67.4%. Lettuce had the highest reduction (67.4 ± 7.3%), whereas ssamchoo had the lowest reduction (40.6 ± 12.9%). Spinach and crown daisy showed no significant difference in their reductions. Based on reduction by method, running water (77.0 ± 18.0%) and boiling (59.5 ± 31.2%) led to the highest reduction, whereas detergent (43.7 ± 14.5%) led to the lowest reduction. The reductions of chlorfenapyr, diniconazole, indoxacarb, fludioxonil, pyraclostrobin, and lufenuron in the leafy vegetables were lower with blanching and boiling than with other methods (*p* < 0.05). These results highlight the importance of thoroughly washing leafy vegetables to lower the intake of pesticide residues before cooking.

## 1. Introduction

Despite their toxicity, pesticides are widely used to protect crops against insects, weeds, fungi, and other pests. Accordingly, pesticides are indispensable for food productivity and quality [1]. The application of pesticides may result in residues on vegetables, and these specified derivatives can induce adverse health effects (acute and chronic effects, such as reproductive harm, carcinogenicity, neurological toxicity, and cell dysplasia) [2,3]. Therefore, excessive consumption of residual pesticides via raw or processed vegetables is dangerous for consumers and thus warrants an effective removal strategy.

Globally, studies have been conducted to derive methods that can be utilized for pesticide residue removal from various types of vegetables. Many studies have used methods, such as peeling/trimming, washing/rinsing, soaking, and blanching/boiling, mainly for fruits and fruiting vegetables [4,5,6,7,8,9,10,11,12]. Tomatoes, oranges, cucumbers, and strawberries have been mainly employed in these studies. However, as these fruits can be peeled and washed relatively easily, it is comparatively simple to remove pesticide residues on these fruits compared to those on vegetables, especially leafy vegetables. Notably, the pesticides analyzed in these studies were limited to those used in fruits and fruiting vegetables. The removal of pesticide residues from the outer skin of certain fruits and fruiting vegetables by peeling or trimming is reported to be the most efficient approach to reduce pesticide residues [13]. However, it is difficult to apply these methods to leafy vegetables, as they cannot be peeled or trimmed. Therefore, the removal method of various pesticides from vegetables must be investigated. As leafy vegetables cannot be peeled, they are relatively difficult to clean; only attached dust, insects, and foreign substances can be removed. In addition, owing to the large surface area of these vegetables, pesticide residues are likely to remain on their surfaces [14].

Leafy vegetables are used in various cuisines worldwide. Hence, an efficient method is required to remove pesticide residues from leafy vegetables in households. Among all methods, washing (tap water) is known as the most common and efficient method of pesticide residue removal in general households [15]. However, as many pesticides are hydrophobic, washing with tap water is inefficient for pesticide residue removal [16]. Cooking is known to be effective at removing some volatile pesticides but is ineffective at removing less volatile pesticides [11]. Therefore, depending on the characteristics of pesticides, various removal methods should be used. In particular, a removal method suitable for the characteristics of pesticides with a high frequency of use and high detection rate is necessary [13].

Several studies investigated the removal of pesticide residues from leafy vegetables. For example, washing spinach with tap water removed 0–48% of boscalid, deltamethrin, iprodione, mancozeb, and propamocarb [17]; washing Chinese cabbage with electrolyzed water removed 32–38% of chlorpyrifos, prothiofos, and deltamethrin; and blanching (5 min at 88 °C) spinach removed 0–72% of boscalid, deltamethrin, iprodione, mancozeb, and propamocarb [17,18]. However, studies using these methods, particularly washing with water, are scarce and are limited to only a few leafy vegetables.

**Figure 1:**
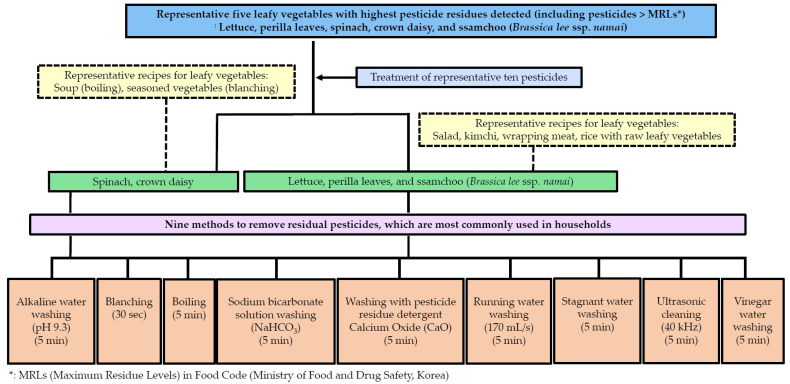
Sampling and processing scheme.

In general, different methods are used to remove pesticide residues, including washing or blanching, in South Korea and other countries. Accordingly, studies comparing the effectiveness of removing various pesticide residues from various leafy vegetables are warranted. The objective of this study was to evaluate the effectiveness of various methods used in households to remove pesticide residues from agricultural products. Agricultural products, particularly leafy vegetables, were selected based on long-term research results obtained in the largest production area of leafy vegetables in South Korea and Food Safety Management Guidelines for 2021: lettuce, perilla leaves, spinach, crown daisy, and ssamchoo (*Brassica lee* ssp. *namai*) (Figure 1). The pesticides used were azoxystrobin, chlorantraniliprole, chlorfenapyr, diniconazole, fludioxonil, imidacloprid, indoxacarb, lufenuron, pyraclostrobin, and thiamethoxam [19,20].

Leafy vegetables are widely consumed in Asia in (1) salad, (2) kimchi, (3) seasoned vegetables, (4) meat wrapping, and (5) vegetable soup. Herein, nine pesticide residue removal methods were selected (alkaline electrolyzed water washing, blanching, boiling, sodium bicarbonate solution washing, washing with detergent, tap water washing-running water, tap water washing-stagnant water, ultrasonic cleaning, and vinegar water washing) for comparison (Figure 1).

To the best of our knowledge, the removal efficiency of the selected pesticides in vegetables, especially leafy vegetables, remains largely unknown. The aim of this study was to determine the effectiveness of nine pesticide residue removal methods on the removal of pesticide residues from leafy vegetables.

## 2. Materials and Methods

### 2.1. Standards, Reagents, and Materials

The following ten pesticides with high detection rates on leafy vegetables were selected: azoxystrobin, chlorantraniliprole, chlorfenapyr, diniconazole, fludioxonil, imidacloprid, indoxacarb, lufenuron, pyraclostrobin, and thiamethoxam (Table 1) [19,20].

#### 2.1.1. Pesticides

The pesticides were purchased as three commercially available formulations (suspension concentrate; SC, water-dispersible granule; WG, and emulsifiable concentrate; EC). Diniconazole (SC, 5% active ingredient) was purchased from Dongbang agro Co., Ltd. (Seoul, South Korea). Chlorfenapyr (SC, 10% a.i.) was purchased from Shin Young agro Co., Ltd. (Seoul, South Korea). Imidacloprid (SC, 8% a.i.) was obtained from Bayer Crop Science (Seoul, South Korea). Thiamethoxam (WG, 10% a.i.), chlorantraniliprole (SC, 2.7% a.i.), fludioxonil (SC, 20% a.i.), and lufenuron (EC, 5% a.i.) were purchased from Syngenta Korea (Seoul, South Korea). Indoxacarb (WG, 5% a.i.) was purchased from Hanearl Science (Gangwon-do, Taebaek-si, South Korea). Azoxystrobin (SC, 21.7% a.i.) and pyraclostrobin (SC, 20% a.i.) were purchased from Chunjiinbiotec (Gangwon-do, Taebaek-si, South Korea).

#### 2.1.2. Standards

Lufenuron (purity: 100%), the pesticide standard, was provided by the Institute of Kemidas (Gyeonggi-do, Gunpo-si, South Korea). The purities of the nine pesticide standards (azoxystrobin, chlorantraniliprole, chlorfenapyr, diniconazole, fludioxonil, imidacloprid, indoxacarb, pyraclostrobin, and thiamethoxam) ranged from 98 to 100%; these standards were obtained from Accustandard (New Haven, CT, USA).

#### 2.1.3. Reagents

The extraction salt kit (4 g MgSO_4_, 1 g NaCl, 0.5 g disodium citrate sesquihydrate, and 1 g trisodium citrate dehydrate) and dispersive solid phase extraction (dSPE) kit (25 mg primary secondary amine and 150 mg MgSO_4_) were purchased from Chromatific (Heidenrod, Germany). Acetonitrile, methanol (Merck, Darmstadt, Germany), formic acid (purity: 99%) (Wako, Osaka, Japan), and ammonium acetate (purity: 99%) (Sigma-Aldrich, St. Louis, MO, USA) were of liquid chromatography grade. Vegetable detergent (CaO, 100%), alkaline water (water 99.99%, calcium hydroxide 0.005%, magnesium hydroxide 0.005%) (pH 9.3), vinegar, and sodium bicarbonate were obtained from Ecobiotec Co., Ltd. (Gyeonggi-do, Hwaseong-si, South Korea), Auskorea Co., Ltd. (Gyeonggi-do, Seongnam-si, South Korea), Daesang Corp. (Seoul, South Korea), and LG Household & Health Care Ltd. (Seoul, South Korea), respectively.

### 2.2. Sample Preparation and Washing Treatments

The following five leafy vegetables with the highest rate of pesticide detection were selected: lettuce, perilla leaves, spinach, crown daisy, and ssamchoo [19,20]. The samples were randomly purchased from the Gakhwa Agricultural Products Wholesale Market (Gwangju, South Korea) in 2021. Samples of the five leafy vegetables were pre-analyzed and determined to be free of previous residues prior to the experiment and stored at 4 °C prior to analysis. The pesticides used to prepare the contaminated samples were administered according to the doses recommended by the manufacturer. The recommended doses were 20 mL/20 L for diniconazole and indoxacarb and 10 mL/20 L for chlorfenapyr, azoxystrobin, imidacloprid, chlorantraniliprole, fludioxonil, and lufenuron. The recommended amounts of thiamethoxam and pyraclostrobin were 10 g/20 L and 6.7 g/20 L, respectively. The concentration of the 10 pesticides in the mixed solution ranged from 13.5–108.5 mg/L. Leafy vegetables were soaked in 20 L of mixed pesticide and treated for 10 s to ensure even application of the pesticide. Contaminated leafy vegetables were air-dried in a fume hood for 15 h at room temperature. Subsequently, 100 g of the contaminated leafy vegetables was randomly collected to detect the initial residual amount. The contaminated samples were washed using nine methods. The treatment time was set to 5 min, which is the mid-point of the treatment time used in previous studies, and was unified, except for blanching (30 s), to compare the washing effect [13]. Each process was repeated five times.

(a) Running tap water: Samples (100 g) were rinsed under running tap water for 5 min. The running rate of the tap water was controlled at 170 mL/s.
(b) Stagnant tap water/alkaline water: Samples (100 g) were soaked in a bucket containing 2 L of stagnant tap water and alkaline water for 5 min.
(c) Ultrasonic cleaning: Samples (100 g) were placed in an ultrasonic cleaner bath (Bransonic CPX8800H-E, Branson, MO, USA) containing stagnant water (2 L). The ultrasonic cleaner was maintained at 40 kHz for 5 min (the frequency of commercially available household ultrasonic cleaners).
(d) 5% vinegar, 2% sodium bicarbonate, and vegetable detergent in water: Samples (100 g) were soaked in a bucket containing 2 L of 5% vinegar, 2% sodium bicarbonate, and vegetable detergent (1.5 g/2 L) in water for 5 min.
(e) Blanching/boiling: Samples (100 g) were placed into a bucket and 2 L of boiling water (100 °C) for 30 s and 5 min, respectively.

### 2.3. Extraction and Analysis of the Pesticide Residues

The extraction was performed using the QuEChERS (Quick, Easy, Cheap, Effective, Rugged, Safe) method [21]. Briefly, the washed samples (100 g) were homogenized and processed using a blender. Thereafter, 10 g of the sample was placed in 50 mL centrifuge tubes and mixed with 10 mL of acetonitrile. The tube was sealed and vigorously shaken in a shaker (VIBA X.30, Collomix, Gaimersheim, Germany) for 1 min. The extraction salt kit was subsequently added. The mixture was shaken vigorously for 1 min and then centrifuged (Avanti J-15R, Beckman Coulter, Brea, CA, USA) at 4000× *g* for 10 min. A 1 mL aliquot of the supernatant was transferred to a 2 mL centrifuge tube containing a dSPE kit. The 2 mL centrifuge tube was then shaken for 1 min and centrifuged (Microfuge 20R, Beckman Coulter, Brea, CA, USA) for 5 min at 10,000× *g*. Finally, the supernatant was filtered through a 0.2 μm membrane into a chromatography vial for analysis.

### 2.4. Chromatographic Analysis

#### 2.4.1. Gas Chromatography-Tandem Mass Spectrometry (GC-MS/MS) Analysis

The pesticides (chlorfenapyr, diniconazole, and indoxacarb) were analyzed using an Agilent 7000D GC/TQ with a 7890 B gas chromatograph and a 7693A autosampler (Agilent Technologies, Santa Clara, CA, USA). Chromatographic separation was achieved on a DB-5MS UI column (0.25 mm I.D. × 30 m, 0.25 μm) (Agilent Technologies, Santa Clara, CA, USA). The oven temperature program was as follows: 200 °C hold for 0.1 min, increased to 250 °C at a rate of 40 °C/min, and increased to 300 °C at a rate of 60 °C/min and hold for 5 min. One microliter of the sample was injected in splitless mode. The total running time was 7.18 min. Helium gas was used as the carrier gas at a constant flow rate of 1.5 mL/min. Triple quadrupole MS was applied in dynamic multiple reaction monitoring mode with electron ionization at 70 eV. The GC-MS/MS parameters are summarized in Table 2. The ion source and transfer line temperatures were set at 250 and 280 °C, respectively. The MassHunter quantitative analysis software (version 10.1) (Agilent Technologies, Santa Clara, CA, USA) was used for data processing.

#### 2.4.2. Liquid Chromatography-Tandem Mass Spectrometry (LC-MS/MS) Analysis

The pesticides (azoxystrobin, chlorantraniliprole, fludioxonil, imidacloprid, lufenuron, pyraclostrobin, and thiamethoxam) were analyzed using a nanospace NASCA (OSAKA SODA, Tokyo, Japan) liquid chromatograph with a QTRAP 4500 detector (AB Sciex, Framingham, MA, USA). The target pesticides were separated on a CAPCELL CORE C18 column (2.1 mm I.D. × 150 mm, 2.7 μm) (OSAKA SODA, Tokyo, Japan) maintained at 40 °C. The mobile phase was 5 mM ammonium acetate in water with 0.1% formic acid (phase A) and 5 mM ammonium acetate in methanol with 0.1% formic acid (phase B). The following gradient was employed: 95:5 (A:B) (0–1 min), 40:60 (1–3 min), 0:100 (3–13 min), and held for 18 min, and finally maintained at 95:5 (18.1–25 min). The flow rate was 200 μL/min. Two microliters of the final extracted sample solution were injected into the system. The analysis time was 26.05 min. Quantification and identification of the target compounds were carried out in multiple reaction monitoring mode. Electrospray ionization was conducted in positive ion mode (ESI+) and negative ion mode (ESI−) at capillary voltages of 5500 and −4500 V, respectively, and evaporation of solvents with synthetic air at 450 °C. Table 2 summarizes the LC-MS/MS parameters.

### 2.5. Method Validation

The analytical method was validated according to the SANTE/12682/2019 guidelines on validation procedures for pesticide residue analysis in food and feed [22]. The limits of detection (LODs) and limits of quantitation (LOQs) were estimated using signal-to-noise (S/N) ratios of 3 and 10, respectively. The analytical method was validated for each matrix, and the linearity of the matrix-matched calibration curve was determined at five concentrations (0.01, 0.025, 0.05, 0.075, and 0.1 mg/kg). Recovery was estimated at three concentrations (0.01, 0.05, and 0.1 mg/kg) by spiking ten standard pesticides into a blank sample.

### 2.6. Statistical Analysis

All data analyses were performed using SPSS Statistics, Version 27.0 (IBM, Armonk, NY, USA). For each washing method, the mean and standard deviation of the data from the repeated experiments were determined. The significance of all data was determined using ANOVA, and Tukey’s test was used as a post-hoc analysis technique. Differences between treatments were established at a significance level of *p* < 0.05. Pearson’s correlation test was conducted to estimate the effect of washing methods and properties of pesticide on the decline pattern. Correlation analysis and principal component analysis (PCA) were performed to determine the correlation between the pesticide physicochemical parameters, pesticide percent reduction, and effectiveness of each washing method.

## 3. Results

### 3.1. Method Validation

In general, method validation was performed using each matrix. Table 3 shows the standard curve coefficients (R^2^), average recoveries, and relative standard deviations for the pesticides studied using each matrix. The LOQs for the ten pesticides (azoxystrobin, chlorantraniliprole, chlorfenapyr, diniconazole, fludioxonil, imidacloprid, indoxacarb, lufenuron, pyraclostrobin, and thiamethoxam) were defined as the concentrations produced from a S/N ratio of 10. The estimated LOD and the LOQ were 0.001–0.003 and 0.002–0.009 mg/kg, respectively. The LOQ was lower than the maximum residue limit (MRL) set by the Ministry of Food and Drug Safety of the South Korea (Table 1). Identical linearities with determination coefficients (R^2^ > 0.999) were obtained from matrix-matched calibration of the blank and each matrix. The recovery rates were satisfactory, ranging from 87 to 115%, with an RSD of <8%. The RSD for five leafy vegetables never exceeded 20% according to the acceptance and rejection criteria of the SANTE guidelines. All mean values for recovery were within the acceptable range (70–120%).

### 3.2. Differences in Efficiency by Type of Leafy Vegetable and Removal Method for the Reduction of Pesticide Residues

In this study, ten pesticides with high detection rates were administered to five leafy vegetables. Thereafter, nine removal methods were applied. As various washing methods are applied for various dishes in Asian countries, including China and South Korea, a representative method for removing pesticide residues from leafy vegetables was tested. Since leafy vegetables are intended to be consumed following these treatments, their structure must remain more or less intact to maintain consumer acceptance. Figure S1 depicts images revealing the effect of the treatment on the appearance of the leafy vegetables. Except for blanching and boiling, all methods preserved the structural integrity of the leafy vegetables. Blanched or boiled leafy vegetables are consumed as soup or seasoned vegetables; therefore, consumers could accept them even if their structure is not retained.

The initial residue values for the pesticides found on artificially contaminated samples are outlined in Table S1: lettuce (4.45–34.81 mg/kg), perilla leaves (5.74–42.97 mg/kg), spinach (4.52–37.04 mg/kg), crown daisy (4.02–29.67 mg/kg), and ssamchoo (2.95–27.96 mg/kg). The reductions in the pesticide amount in the five vegetables are summarized in Table 4. The reduction range for the five leafy vegetables was 40.6–67.4%. The average reductions for each sample appeared in the following order: lettuce (67.4 ± 7.3%) > perilla leaves (59.8 ± 10.2%) > spinach (55.1 ± 13.8%) and crown daisy (54.3 ± 11.5%) > ssamchoo (40.6 ± 12.9%). Spinach and crown daisy showed no significant difference in their reductions. Lettuce had the highest reduction (57.5% (detergent)–82.5% (running water)), whereas ssamchoo had the lowest reduction (28.0% (NaHCO_3_)–59.7% (running water)). Overall, the reductions for each method were as follows: running water (77.0 ± 18.0%), boiling (59.5 ± 31.2%), alkaline water (56.4 ± 18.0%), blanching (54.9 ± 25.9%), ultrasonic cleaning (52.8 ± 18.7%), NaHCO_3_ (52.0 ± 19.2%), stagnant water (51.4 ± 16.4%), vinegar (51.2 ± 18.3%), detergent (43.7 ± 14.5%). Washing with running water led to the highest removal efficiency among all methods, whereas washing with detergent led to the lowest removal efficiency.

### 3.3. Comparison of the Removal Efficiency of Each Pesticide Residue

**Figure 2:**
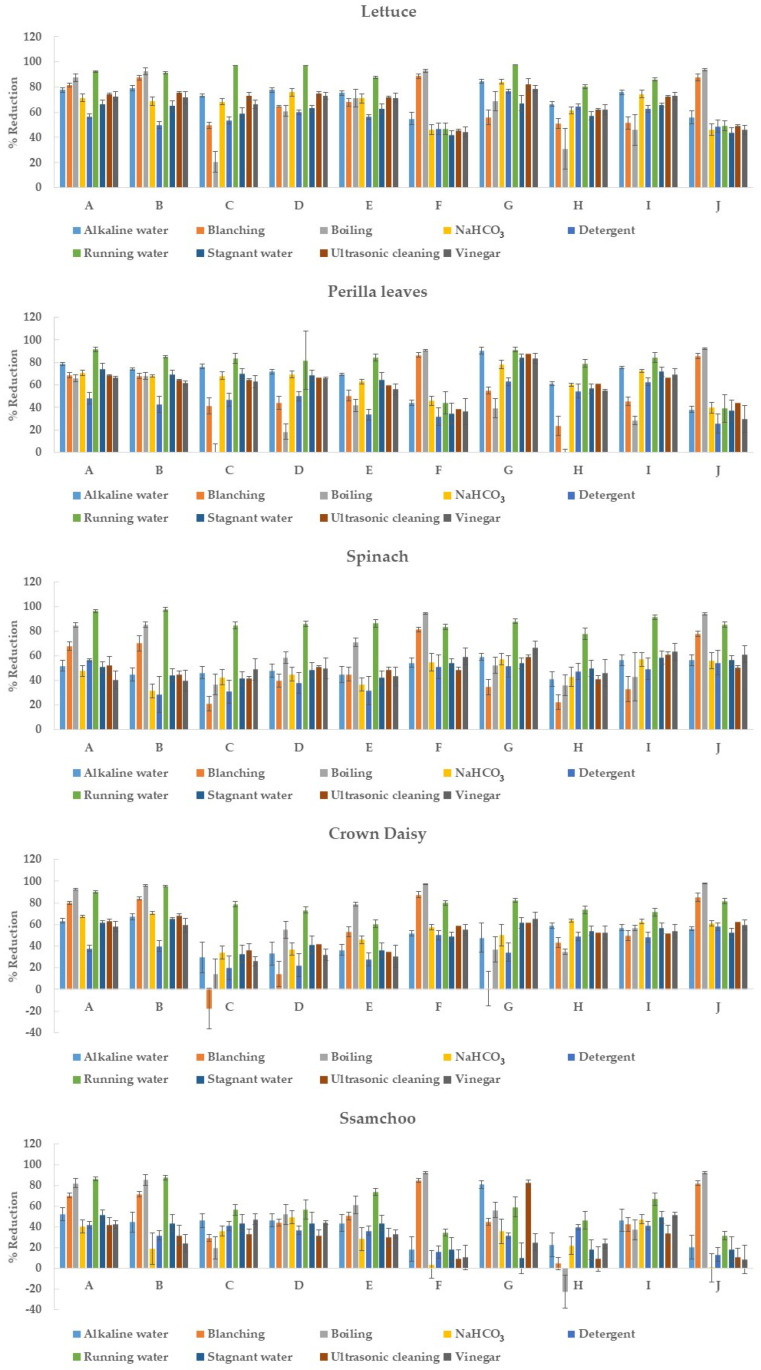
Comparison of removal efficiency of each pesticide in five leafy vegetables (n = 5). A: azoxystrobin, B: chlorantraniliprole, C: chlorfenapyr, D: diniconazole, E: fludioxonil, F: imidacloprid, G: indoxacarb, H: lufenuron, I: pyraclostrobin, and J: thiamethoxam.

The reductions of each pesticide in the five leafy vegetables using the nine methods are shown in Figure 2. Table S1 presents the numerical results. The initial residual values of pesticides found in artificially contaminated samples were 27.96–42.97 mg/kg (azoxystrobin), 2.95–5.74 mg/kg (chlorantraniliprole), 11.54–16.27 mg/kg (chlorfenapyr), 10.95–15.56 mg/kg (diniconazole), 21.64–39.28 mg/kg (fludioxonil), 8.54–16.03 mg/kg (imidacloprid), 8.72–20.76 mg/kg (indoxacarb), 4.88–9.04 mg/kg (lufenuron), 16.65–26.95 mg/kg (pyraclostrobin), and 9.27–17.35 mg/kg (thiamethoxam). As pesticide treatment was not performed in the field, the initial pesticide residues exceeded the MRL in most cases.

The experimental conditions were not identical to those of the crop-growing environment, which served as a limitation. Therefore, it was difficult to treat pesticides according to the growth stage, harvest time, and frequency of use of crops, and these changes could not be reflected in the results. Although we excluded these factors, the pesticides were assumed to be administered at their maximum value. Furthermore, this study sought to identify the cleaning effect of various cleaning methods.

The average reductions of each pesticide appeared in the following order: azoxystrobin (66.2 ± 7.6%) > chlorantraniliprole (63.3 ± 11.5%) > indoxacarb (61.2 ± 14.1%) > pyraclostrobin (58.1 ± 8.2%) > thiamethoxam (54.0 ± 15.2%), imidacloprid (53.6 ± 13.7%), fludioxonil (53.5 ± 11.0%), and diniconazole (53.3 ± 12.9%) > chlorfenapyr (46.0 ± 13.8%) and lufenuron (45.1 ± 16.0%). For the overall reduction by pesticide, azoxystrobin had the highest reduction (66.2%), whereas lufenuron had the lowest reduction (45.1%). A reduction difference of approximately 20% was found between the highest and lowest values for each pesticide. The reduction efficiencies of thiamethoxam, imidacloprid, fludioxonil, and diniconazole were not significantly different. Furthermore, lufenuron and chlorfenapyr were not significantly different.

### 3.4. Statistical Analysis of the Physicochemical Parameters and Removal Efficiency of Ten Pesticides

**Figure 3:**
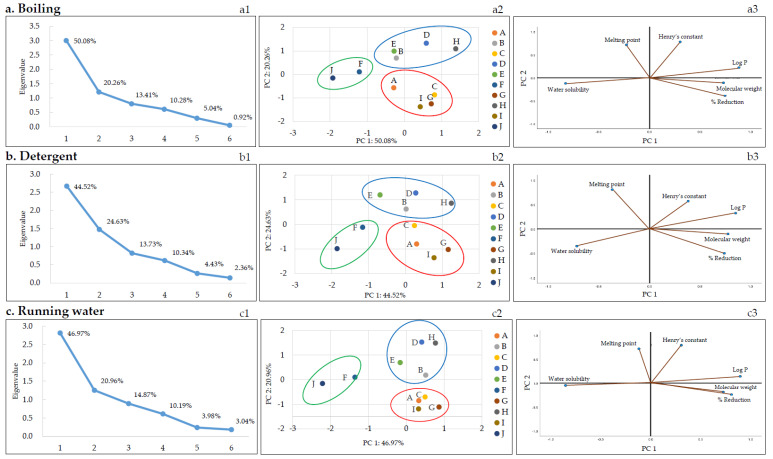

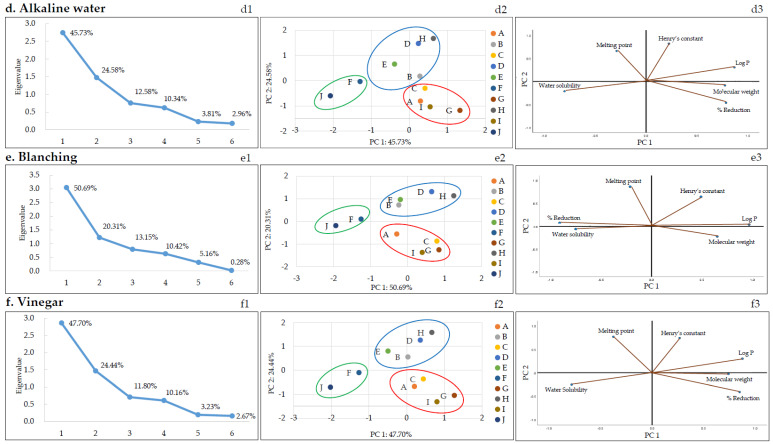

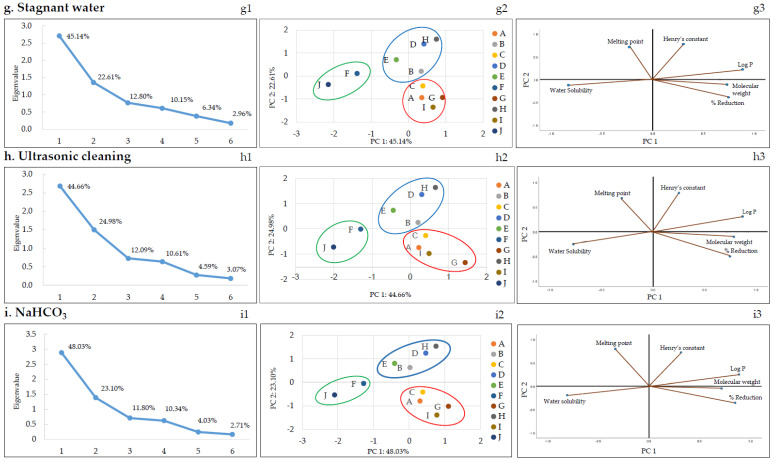
The plots of PCA: The scree (**a**–**i1**), score (**a**–**i2**), and loading (**a**–**i3**) plots. A: Azoxystrobin, B: Chlorantraniliprole, C: Chlorfenapyr, D: Diniconazole, E: Fludioxonil, F: Imidacloprid, G: Indoxacarb, H: Lufenuron, I: Pyraclostrobin, and J: Thiamethoxam.

According to the correlation analysis (Table 5), the dominant physicochemical parameter affecting the reduction of pesticide residues during thermal processing (blanching and boiling) was log *P* (negative correlation) and that for the running water washing method was water solubility (negative correlation). PCA was performed to better understand the correlation between various physicochemical parameters of pesticides and pesticide reduction in each of the nine removal methods. Figure 3 shows the PCA results of the nine methods and their characteristic results. Of note, the relationship between the molecular weight, polarity (log *P*), water solubility, melting point, Henry’s constant of the pesticides, and reduction is discussed below for each method of pesticide residue removal. Score plots were obtained for each removal method using PCA. The first and second principal components (PC1 and PC2, respectively) were selected according to the Kaiser’s rule of selecting principal components with eigenvalues greater than 1 (Figure 3a–i1). PC1 and PC2 were further analyzed.

Score plots and loading plots revealed that the PC1 and PC2 accounted for more than 68% of the total variance in PCA. The two significant principal components accounted for a proportion of the total variation: running water: 67.93% (PC1: 46.97% and PC2: 20.96%), boiling: 70.3% (PC1: 50.08% and PC2: 20.26%), detergent: 69.14% (PC1: 44.52% and PC2: 24.63%), alkaline water: 70.3% (PC1: 45.73% and PC2: 24.58%), blanching: 71.0% (PC1: 50.69% and PC2: 20.31%), vinegar: 72.14% (PC1: 47.70% and PC2: 24.44%), stagnant water: 67.76% (PC1: 45.14% and PC2: 22.61%), ultrasonic cleaning: 69.64% (PC1: 44.66% and PC2: 24.98%), and NaHCO_3_: 71.12% (PC1: 48.03% and PC2: 23.10%). The pesticides were categorized based on the scores and loadings and grouped into three clusters, as shown in Figure 3a–i2. Each group consisted of the following pesticides: first pesticides group (imidacloprid and thiamethoxam), second pesticides group (chlorantraniliprole, diniconazole, fludioxonil, and lufenuron), and third pesticides group (azoxystrobin, chlorfenapyr, indoxacarb, and pyraclostrobin). Imidacloprid and thiamethoxam, classified into the first group, displayed similar reduction patterns in all methods based on Figure 2 and Figure 3. The first group had log *P* less than 1 and high water solubility, and the second and third groups had log *P* greater than 1 and low water solubility.

## 4. Discussion

### 4.1. Differences in Efficiency Based on the Type of Leafy Vegetable and Removal Method for the Reduction of Pesticide Residues

In this study, the reduction range for five leafy vegetables was 40.6–67.4% (Table 4). Previous studies revealed the effectiveness of pesticide residue removal from fruits and fruiting vegetables. When tomatoes and cucumbers were washed for 1 min, over 83–100% of the pesticide residues (dimethoate and profenofos) were removed. When okra (*Abelmoschus esculentus*) was washed for 1 min, malathion was almost completely removed. Up to 70–100% of azoxystrobin, acrinathrin, and kresoxim-methyl were removed when zucchini was washed (intensive) [23,24]. In China, when the kumquat (*Citrus japonica*) was washed with tap water for 5 min, the average reduction of the 10 pesticides (chlorpyrifos, myclo-butanil, tebuconazole, bifenthrin, lambda-cyhalothrin, beta-cypermethrin, esfenvalerate, difenoconazole, imidacloprid, and acetamiprid) was 25%. Furthermore, when spinach was washed in the same manner, the average removal efficiency was 11%. The reduction of the pesticide amount in spinach was lower than that in kumquat for the ten pesticides (chlorpyrifos, myclobutanil, tebuconazole, bifenthrin, lambda-cyhalothrin, beta-cypermethrin, esfenvalerate, difenoconazole, acetamiprid, and imidacloprid) [25]. The removal efficiency of leafy vegetables was relatively lower than that of fruits, as they are consumed without peeling. Removing the skin of fruits is known to have the greatest pesticide removal efficiency [13]. Peeling mangoes has been reported to completely remove fenthion, dimethoate, cypermethrin, and fenvalerate [26]. Based on a comprehensive review of the results of this study and other studies, it is concluded that leafy vegetables tend to have lower pesticide reduction than fruits and fruiting vegetables.

Spinach washed with running water (87.8%) and ssamchoo treated with NaHCO_3_ (28.0%) showed a three-fold difference in reduction (Table 4). Overall, leafy vegetables (perilla leaves and lettuce; average reduction, 63.6%) with a large leaf surface area had a higher reduction than those with a small surface area (spinach and crown daisy; average reduction, 54.7%) (*p* < 0.05); this is because each removal method is considered to have high efficiency over a large area [27]. However, although ssamchoo is a leafy vegetable with broad leaves, such as lettuce and perilla leaves, it has a different aspect ratio (lowest reduction on average). This finding indicates that the reduction may differ depending on the area and surface characteristics (wax amounts on the cuticle, curvature of the surface, etc.) of each agricultural product, despite the similar shape of leafy vegetables. Ssamchoo was developed in 1998 through the interspecies hybridization of Chinese cabbage and cabbage. Accordingly, ssamchoo is a new vegetable with completely different ecological characteristics, shapes, and genetic compositions from Chinese cabbage or cabbage [28].

Washing is the most effective method for removing pesticide residues from agricultural products in households and commercial processing [17]. The removal efficiency of pesticide residues by washing is affected by various elements (including the nature of sample, such as thickness, type, wax amounts on the cuticle, and surface area; pesticide characteristics; retention time of various pesticide residues; and washing methods) [11,29,30,31]. Despite these various factors, washing is the most effective treatment for leafy vegetables. In a study by Kim, washing crown daisy with a commercial detergent (0.5%) was found to reduce pesticide residues by 71.3–87.9%, owing to their ability to dissolve non-polar pesticides. Furthermore, when washed with NaCl (1%), vinegar (5%), and charcoal (1%), the reductions were 80.4–87.3%, 76.9–89.0%, and 78.5–88.2%, respectively. However, the reduction was lower than that of washing with water (80.2–90.5%) [32].

The reductions caused by the other eight methods were lower than those caused by washing with running water. The reduction efficiencies of alkaline water, blanching, vinegar, NaHCO_3_, stagnant water, and ultrasonic cleaning were approximately 51.2–56.4%. The removal efficiency was in the following order: running water > boiling > vinegar, stagnant water, and NaHCO_3_ > detergent (*p* < 0.05). Washing with detergent led to the lowest removal efficiency (43.7 ± 14.5) among all methods (Table 4). Other studies reported results different from those found in this study. Washing cucumber with micron calcium solution was more effective than washing with tap water, alkaline electrolyzed water (pH 10.50 and 12.35), or sodium bicarbonate. Washing with micron calcium solution for 20 min caused a greater loss of ten pesticides in cucumber, leading to a removal efficiency of 60% on average [25]. This difference might be due to the differences in agricultural products, processing times, and pesticides.

In Asian food culture, many foods (vegetable soup, seasoned vegetables) are consumed following the boiling or blanching of leafy vegetables. Residual pesticides in agricultural products are considered non-residue, as they decompose upon heating and cooking. However, according to the results of this study, blanching and boiling caused 54.9% and 59.5% reductions, respectively, which were lower than those induced by washing (*p* < 0.05). This finding highlights the importance of cooking after sufficient washing of leafy vegetables.

### 4.2. Comparison of the Removal Efficiency of Each Pesticide Residue

As mentioned earlier, ssamchoo had the lowest pesticide reduction among the five leafy vegetables, which might be due to the following two pesticides: imidacloprid (3.5–92.1%, average 31.8%) and thiamethoxam (0.2–92.0%, average 30.7%). Imidacloprid and thiamethoxam also had relatively low reductions in the perilla leaves and lettuce. For other pesticides, washing with running water was the most effective. However, imidacloprid and thiamethoxam had significantly higher removal efficiencies with boiling and blanching than those with other methods (Figure 2). This finding can be explained by the fact that an increase in temperature during heating affects the hydrolysis of pesticide compounds [33]. In addition, the removal patterns of the two pesticides were similar for the five leafy vegetables; this is due to the characteristics of each pesticide component. Imidacloprid and thiamethoxam are neonicotinoid-based insecticides, with higher water solubility compared to that of other pesticides, and are non-volatile substances with a log *P* value of <1 and high polarity.

The reductions in chlorfenapyr and lufenuron contents using the blanching and boiling methods tended to be lower than those of other pesticides (*p* < 0.05). In crown daisy, the residual amounts of chlorfenapyr after blanching were higher than their initial values. In addition, the residual level of lufenuron increased in perilla leaves and ssamchoo after boiling. Similarly, Yang et al. reported that the residual level of acetamiprid increased in green chilis after boiling and stir-frying (reduction: approximately 80%) [34]. This finding might be due to the concentration of pesticides as a result of moisture evaporation via heating in an open environment [35]. In a study by Lozowicka et al., the concentration of most pesticides was significantly reduced after 5 min of the boiling process used to prepare strawberry jam. However, pyrethroids (alpha-cypermethrin, deltamethrin, and lambda-cyhalothrin) had a processing factor (PF) of 1 or more (when the PF is 1 or more, the residual pesticide amount is higher than the initial amount). The solubility of alpha-cypermethrin, deltamethrin, and lambda-cyhalothrin was low, at 0.004, 0.0002, and 0.005 mg/L, respectively [36]. The authors judged that the removal of pesticide residues during the heat treatment process would be influenced by the strong adsorption of the pesticides onto plant tissues and the solubility of the pesticides in water. Similarly, in this study, the residual amount of the pesticides chlorfenapyr and lufenuron was higher than the initial amount in the boiling process, and these pesticides had relatively low solubility (0.112 mg/L and 0.046 mg/L, respectively) compared to other pesticides (Table 1). Therefore, it is judged that the characteristics of the pesticides, such as the solubility as well as evaporation of water during the boiling process, may have influenced the residual amount.

Diniconazole had the lowest reduction after lufenuron and chlorfenapyr (*p* < 0.05). Diniconazole was characteristically and mainly detectable in ssamchoo [20]. Therefore, diniconazole must be removed from ssamchoo prior to consumption. When ssamchoo grows too large, it is less commercialized. Therefore, diniconazole is used to control the growth of ssamchoo to a moderate size. Diniconazole is a broad-spectrum triazole fungicide that acts as a plant growth regulator, decreasing the height and leaf area in bean plants when applied to roots [37,38]. In this study, the reduction of diniconazole contents in ssamchoo using different methods showed the following trend: running water (56.7%) and boiling (51.9%) > detergent (36.2%) > ultrasonic cleaning (31.6%) (*p* < 0.05). Overall, the reduction in pesticide residues in ssamchoo was low; washing with running water and boiling proved to be the most effective, especially for the removal of diniconazole from ssamchoo. The ultrasonic method and detergents led to the lowest reductions. Therefore, both washing and boiling are recommended for ssamchoo.

The reduction amounts of chlorfenapyr, diniconazole, indoxacarb, fludioxonil, pyraclostrobin, and lufenuron in all leafy vegetables were low after blanching and boiling (*p* < 0.05). Crown daisy and spinach are consumed as vegetables after blanching in boiling water or as soup after boiling for a long time. Therefore, crown daisy and spinach must be cooked after washing to sufficiently remove these pesticides.

In a previous study from 2005 to 2019, the MRL of leafy vegetables in South Korea was highly exceeded for diniconazole and lufenuron. The detection amount of diniconazole in ssamchoo and perilla leaves was 0.4–6.6 mg/kg and 0.7–2.4 mg/kg, and the MRL was 0.3 mg/kg. Furthermore, the detection amount exceeding the MRL (5.0 mg/kg) of lufenuron in spinach and crown daisy was 0.4–3.8 mg/kg and 0.3–2.1 mg/kg [20]. When running water was used for washing, the reduction of diniconazole from ssamchoo and perilla leaves (ssamchoo, 56.7%; perilla leaves, 81.8%) was highest (Table S1). The reduction of lufenuron in these two leafy vegetables (spinach, crown daisy) was also the highest when running water was employed as the washing method (spinach—77.9%, crown daisy—73.4%). In previous studies, leafy vegetables that do not meet the MRL were not harmful to health based on a risk assessment (risk index ranged from 0.001 to 7.6%) [20]. However, washing with running water before ingestion can reduce pesticide residues to levels below the MRL concentration.

### 4.3. Statistical Analysis between Physicochemical Parameters and Removal Efficiency of Ten Pesticides

As mentioned earlier, correlation analysis (Table 5) revealed that the dominant physicochemical parameter affecting the rate of pesticide residue removal during heat treatment (blanching and boiling) was log *P* (negative correlation). As depicted in Table 1, the log *P* values of chlorfenapyr, diniconazole, indoxacarb, fludioxonil, pyraclostrobin, and lufenuron ranged between 3.99 and 5.12, and the log *P* values of azoxystrobin, chlorantraniliprole, imidacloprid, and thiamethoxam ranged between −0.13 and 2.86. Therefore, the pesticide residues for chlorfenapyr, diniconazole, indoxacarb, fludioxonil, pyraclostrobin, and lufenuron, which have relatively high log *P* values, could not be easily removed. Similarly, Nagayama reported that the log *P* value and reduction were inversely proportional based on an analysis of the residual pesticide amount after blanching or making jam using agricultural products collected from the market [39]. Reichman et al. reported volatility changes owing to phase distribution according to Henry’s constant when a pesticide is placed in a wet medium. Therefore, in their soil test, the highest volatilization rate of trifluralin was reported to correspond to the highest Henry’s constant [40]. Similarly, in a study by Kwon, the reduction in pesticide residues by boiling water was proportional to Henry’s constant of pesticides [27]. However, by comparing the pesticide residue reduction and Henry’s constant, a proportional relationship with Henry’s constant was not found. The dominant variables in the PCA plot were water solubility and Log *P* (Figure 3a–i3). These two variables are the important variables that have the greatest influence on PC1.

## 5. Conclusions

A comparative study was conducted to determine the effectiveness of removing ten pesticide residues from five leafy vegetables artificially contaminated with pesticides using nine removal methods. The reduction range for each method was 43.7–77.0% and that for five leafy vegetables was 40.6–67.4%. Lettuce had the highest reduction (67.4 ± 7.3%), whereas ssamchoo had the lowest reduction (40.6 ± 12.9%). Spinach and crown daisy showed no significant difference in their reductions. On average, removal using running water (77.0 ± 18.0%) and boiling (59.5 ± 31.2%) led to the highest reduction, whereas using detergent (43.7 ± 14.5%) led to the lowest reduction. The reductions in chlorfenapyr, diniconazole, indoxacarb, fludioxonil, pyraclostrobin, and lufenuron in the five leafy vegetables were lower following blanching and boiling than that following the other methods. High log *P* values (3.99–5.12, which is greater than 1) were considered to be one of the causes of this result. Therefore, to increase the removal efficiency of these pesticide residues, the vegetable must be boiled and blanched after sufficient washing. Further research on various leafy vegetables associated with high consumption of pesticides should be conducted based on our results, as leafy vegetables have a high risk of pesticide residue contamination.

## Figures and Tables

**Table 1:** The main properties of ten pesticides.

Pesticides ^(1)^ (CAS No.)	Leafy Vegetables ^(2)^	MRL ^(3)^ (mg/kg)	Category	Molecular Weight	Water Solubility at 20 °C (mg/L)	Log *P* ^(4)^	Melting Point (°C)	Henry’s Constant at 25 °C (Pa m^3^ mol^−1^)
Azoxystrobin (131860-33-8)	Perilla leaves, aster scaber, danggi leaf (Korean angelica root leaf)	20	Fungicide	403.4	6.7	2.5	116	7.40 × 10^−9^
Chlorantraniliprole (500008-45-7)	Spinach, chives	5.0	Insecticide	483.2	0.88	2.86	209	3.2 × 10^−9^
Chlorfenapyr (122453-73-0)	Crown daisy, lettuce, danggi leaf	5.0	Acaricide, Insecticide	407.6	0.112	4.83	101	5.81 × 10^−4^
Diniconazole (83657-24-3)	Ssamchoo (*Brassica lee* ssp. *namai*), perilla leaves, crown daisy	0.3	Fungicide	326.2	4	4.3	145	4.00 × 10^−2^
Fludioxonil (131341-86-1)	Crown daisy	15	Fungicide	248.2	1.8	4.12	199.8	5.40 × 10^−5^
Imidacloprid (138261-41-3)	Perilla leaves, pepper leaves	3.0	Insecticide	255.7	610	0.57	144	1.7 × 10^−10^
Indoxacarb (173584-44-6)	Spinach, danggi leaf	3.0	Insecticide	527.8	0.2	4.65	88.1	6.00 × 10^−5^
Lufenuron (103055-07-8)	Spinach, crown daisy, lettuce, mustard green	5.0	Acaricide, Insecticide	511.2	0.046	5.12	169.1	3.41 × 10^−2^
Pyraclostrobin (175013-18-0)	Perilla leaves, chives	15	Fungicide	387.8	1.9	3.99	63.7	5.31 × 10^−6^
Thiamethoxam (153719-23-4)	Crown daisy, curled mallow	5.0	Insecticide	291.7	4100	−0.13	139.1	4.70 × 10^−10^

^(1)^ Pesticides with high nonconformity and detection rate in the previous study and Food Safety Management Guidelines for 2021 [19,20]. ^(2)^ Main leafy vegetables with the highest detection of each pesticide in the previous study and Food Safety Management Guidelines for 2021 [19,20]. ^(3)^ MRL of leafy vegetable set by the Ministry of Food and Drug Safety, South Korea [21]. ^(4)^ The values of Log *P* are octanol-water partition coefficient at pH 7, 20 °C.

**Table 2:** Experimental parameters of ten pesticides by GC-MSMS and LC-MSMS.

Pesticide	Retention Time (min)	Precursor Ion (*m/z*)	Product Ion (*m/z*)	Collision Energy (eV)
GC-MSMS				
Chlorfenapyr	2.74	247	227	15
		328	247	15
Diniconazole	2.90	268	136	45
			232	15
Indoxacarb	5.59	203	106	25
			134	15
LC-MSMS				
Azoxystrobin	8.85	404	372	21
			344	35
Chlorantraniliprole	8.62	484	453	27
			286	21
Fludioxonil	9.14	266	229	23
			158	50
Imidacloprid	5.89	256	209	25
			212	17
Lufenuron	12.87	509	339	15
			326	30
Pyraclostrobin	11.48	388	163	39
			194	18
Thiamethoxam	5.57	292	211	17
			132	35

**Table 3:** Regression coefficient (R^2^), LOQs, and average recoveries for ten pesticides in five leafy vegetables (n = 5).

Pesticide	Linearity (R^2^), 0.01–0.1 mg/kg	LOQ (mg/kg)	Average Recovery					
			0.01 mg/kg		0.05 mg/kg		0.1 mg/kg	
			%	%RSD	%	%RSD	%	%RSD
Lettuce								
Azoxystrobin	0.9993	0.004	102.1	3.3	105.7	4.8	102.8	2.3
Chlorantraniliprole	0.9996	0.005	100.5	4.0	100.6	3.4	100.5	3.1
Chlorfenapyr	0.9999	0.003	102.7	3.1	113.1	1.7	103.8	1.4
Diniconazole	1.0000	0.002	105.3	0.7	109.0	0.5	104.8	0.8
Fludioxonil	0.9995	0.005	86.8	2.3	92.7	2.5	90.6	3.0
Imidacloprid	0.9999	0.005	88.0	2.2	91.1	2.1	88.5	2.9
Indoxacarb	0.9993	0.005	104.8	4.1	114.6	2.9	115.4	1.9
Lufenuron	0.9999	0.003	97.6	4.7	102.2	5.1	100.9	4.1
Pyraclostrobin	0.9992	0.009	102.7	1.9	104.5	1.8	103.0	1.9
Thiamethoxam	0.9999	0.002	87.4	3.3	90.0	1.0	87.9	3.2
Perilla leaves								
Azoxystrobin	0.9998	0.006	98.8	6.5	102.1	5.8	96.8	2.8
Chlorantraniliprole	0.9999	0.006	98.6	5.7	96.4	6.9	93.5	4.0
Chlorfenapyr	1.0000	0.004	105.2	6.4	99.5	1.1	99.9	3.5
Diniconazole	1.0000	0.003	102.1	3.2	100.1	1.7	99.0	1.7
Fludioxonil	0.9999	0.009	98.8	5.3	96.2	5.5	103.3	5.1
Imidacloprid	0.9998	0.003	98.1	0.5	96.1	2.2	96.6	1.9
Indoxacarb	0.9999	0.004	98.6	4.6	98.1	3.8	104.2	3.0
Lufenuron	0.9999	0.006	97.2	2.4	96.2	3.3	97.0	4.9
Pyraclostrobin	0.9999	0.005	96.7	2.2	96.9	2.3	100.7	3.5
Thiamethoxam	0.9999	0.006	96.0	1.5	95.1	3.0	95.3	2.0
Spinach								
Azoxystrobin	0.9999	0.002	100.5	1.2	93.0	3.2	96.9	3.8
Chlorantraniliprole	0.9998	0.002	100.4	4.8	95.2	3.4	101.5	2.0
Chlorfenapyr	0.9999	0.004	100.0	5.5	99.0	1.0	101.0	0.7
Diniconazole	0.9997	0.003	103.1	1.3	99.1	1.4	99.6	1.0
Fludioxonil	0.9998	0.003	102.6	1.4	100.3	2.2	94.5	2.7
Imidacloprid	0.9999	0.004	99.0	1.8	102.9	3.3	98.6	1.2
Indoxacarb	0.9994	0.002	99.0	5.0	97.9	3.3	97.6	0.9
Lufenuron	0.9999	0.004	99.7	4.4	101.9	4.2	94.7	2.3
Pyraclostrobin	0.9999	0.003	102.5	3.9	97.8	2.2	104.1	4.2
Thiamethoxam	0.9999	0.002	99.9	1.5	95.9	2.8	100.2	4.7
Crown daisy								
Azoxystrobin	0.9999	0.003	102.6	2.0	101.4	1.9	102.0	1.7
Chlorantraniliprole	0.9999	0.004	95.8	1.9	102.7	1.8	102.6	2.2
Chlorfenapyr	0.9999	0.003	101.8	3.3	101.5	2.2	99.4	0.9
Diniconazole	0.9999	0.002	102.0	0.9	100.2	2.3	102.0	0.6
Fludioxonil	0.9998	0.006	99.5	6.4	102.8	5.3	98.1	4.7
Imidacloprid	0.9999	0.004	97.3	4.0	96.2	4.5	100.8	4.0
Indoxacarb	0.9998	0.007	102.7	5.7	96.1	5.2	100.7	2.7
Lufenuron	0.9999	0.002	88.2	7.5	98.6	1.8	98.0	1.5
Pyraclostrobin	0.9999	0.007	95.1	2.9	95.5	1.2	94.1	2.2
Thiamethoxam	1.0000	0.006	93.2	3.9	96.0	3.0	98.9	1.9
Ssamchoo								
Azoxystrobin	0.9997	0.003	93.2	1.3	95.4	1.9	95.8	4.8
Chlorantraniliprole	0.9999	0.002	95.5	3.5	97.1	4.7	98.4	4.0
Chlorfenapyr	1.0000	0.005	99.9	3.0	99.5	1.6	99.1	1.3
Diniconazole	0.9999	0.006	101.4	1.2	99.7	0.9	98.8	0.6
Fludioxonil	0.9998	0.002	89.7	2.0	103.1	5.1	90.8	0.9
Imidacloprid	0.9999	0.002	97.8	1.9	101.6	3.4	100.3	5.0
Indoxacarb	0.9996	0.006	101.2	4.6	99.1	3.6	100.3	1.4
Lufenuron	1.0000	0.002	96.9	1.8	96.5	1.3	95.5	2.9
Pyraclostrobin	0.9999	0.002	98.6	3.2	99.7	2.7	100.4	3.1
Thiamethoxam	0.9999	0.002	90.2	2.3	93.2	1.6	95.7	1.3

**Table 4:** Reduction of pesticide residues in five leafy vegetables using nine methods (means ± SD, n = 5).

Treatment	% Reduction					
	Lettuce	Perilla Leaves	Spinach	Crown Daisy	Ssamchoo	Mean
Alkaline water	72.0 ± 1.9	67.8 ± 1.2	50.3 ± 4.5	49.9 ± 4.8	42.0 ± 7.9	56.4 ± 18.0 ^bc^
Blanching	68.4 ± 1.8	56.7 ± 3.9	49.3 ± 3.6	47.9 ± 5.4	52.3 ± 2.3	54.9 ± 25.9 ^cd^
Boiling	66.5 ± 4.9	44.0 ± 3.3	65.6 ± 4.3	65.9 ± 2.9	55.4 ± 7.0	59.5 ± 31.2 ^b^
NaHCO_3_	66.7 ± 2.9	63.5 ± 1.8	47.1 ± 5.7	54.8 ± 2.7	28.0 ± 8.6	52.0 ± 19.2 ^d^
Detergent	57.5 ± 2.3	45.8 ± 5.1	43.9 ± 8.3	38.6 ± 4.5	32.6 ± 2.7	43.7 ± 14.5 ^e^
Running water	82.5 ± 1.3	76.3 ± 4.5	87.8 ± 2.1	78.5 ± 0.8	59.7 ± 4.1	77.0 ± 18.0 ^a^
Stagnant water	59.0 ± 2.7	63.1 ± 4.8	50.0 ± 4.6	50.9 ± 3.4	33.7 ± 8.4	51.4 ± 16.4 ^d^
Ultrasonic cleaning	68.0 ± 0.9	62.1 ± 1.0	49.7 ± 2.0	52.9 ± 2.0	31.2 ± 7.4	52.8 ± 18.7 ^cd^
Vinegar	65.7 ± 2.2	58.6 ± 5.2	51.8 ± 6.4	49.1 ± 3.4	30.9 ± 4.5	51.2 ± 18.3 ^d^
Mean	67.4 ± 7.3 ^A^	59.8 ± 10.2 ^B^	55.1 ± 13.8 ^C^	54.3 ± 11.5 ^C^	40.6 ± 12.9 ^D^	-

The different letters indicate significant different (*p* < 0.05) and comparison of means were formed using Tukey’s test.

**Table 5:** Correlation analysis between nine washing methods and properties of pesticide.

Treatment	Pearson’s r				
	Molecular Weight	Water Solubility	Log *P*	Melting Point	Henry’s Constant
Alkaline water	0.627	−0.515	0.475	−0.373	−0.213
Blanching	−0.454	0.512	−0.920 **	0.317	−0.451
Boiling	−0.467	0.476	−0.866 **	0.300	−0.476
NaHCO_3_	0.535	−0.607	0.606	−0.520	0.049
Detergent	0.603	−0.249	0.366	−0.589	0.160
Running water	0.550	−0.672 *	0.573	−0.033	−0.064
Stagnant water	0.495	−0.555	0.469	−0.271	−0.081
Ultrasonic cleaning	0.616	−0.406	0.430	−0.411	−0.180
Vinegar	0.592	−0.539	0.585	−0.592	−0.046

* *p* < 0.05; ** *p* < 0.01.

## Data Availability

Data is contained within the article or supplementary materials and is available upon reasonable request from the corresponding author.

## Supplementary Materials

The following supporting information can be downloaded at: https://www.mdpi.com/article/10.3390/foods11182916/s1, Table S1: The results of nine washing methods for pesticide reduction in five leafy vegetables (*n* = 5). Figure S1: The effect of the treatment on the appearance of the vegetables (spinach and ssamchoo). Note: A: Unwashed, B: Alkaline water washing, C: Blanching, D: Boiling, E: NaHCO_3_, F: Detergent, G: Running water, H: Stagnant water, I: Ultrasonic cleaning, and J: Vinegar.
