# Supplementary material for: Effectiveness of Different Washing Strategies on Pesticide Residue Removal: The First Comparative Study on Leafy Vegetables
Source: Foods. 2022 Sep 19;11(18):2916. doi: 10.3390/foods11182916 (PMC9498324; doi:10.3390/foods11182916)
Supplement: Supplementary file 1 [file foods-11-02916-s001.zip › foods_supplementary_Figure S1_Final .pdf]

Spinach

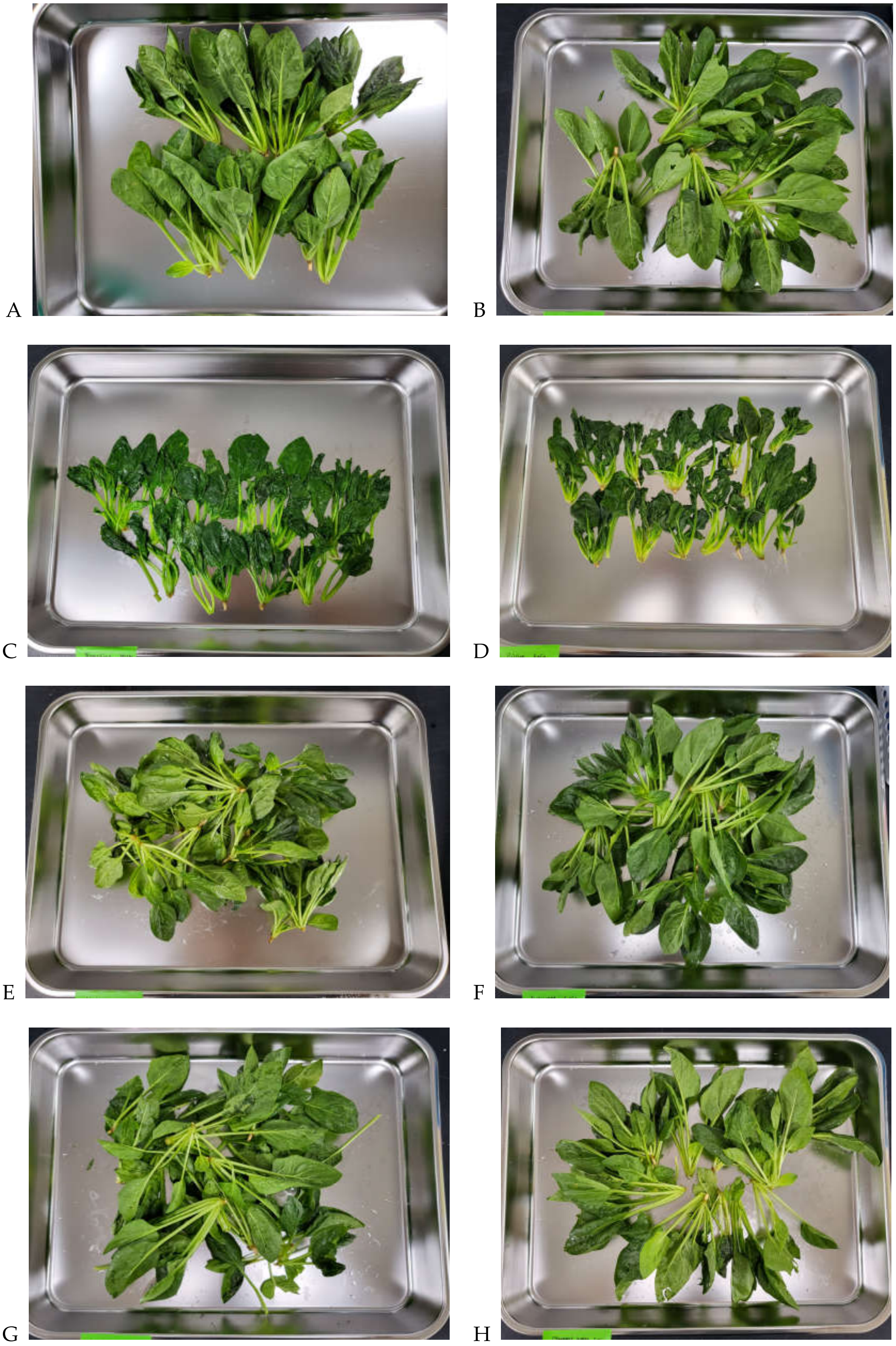

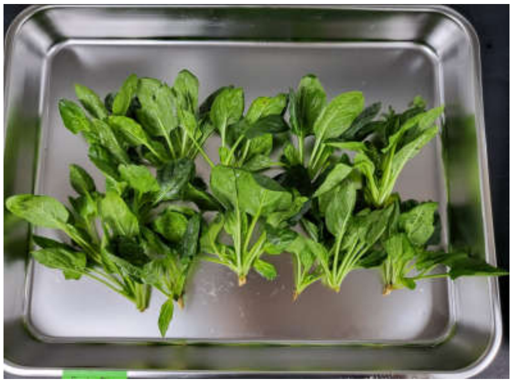
I

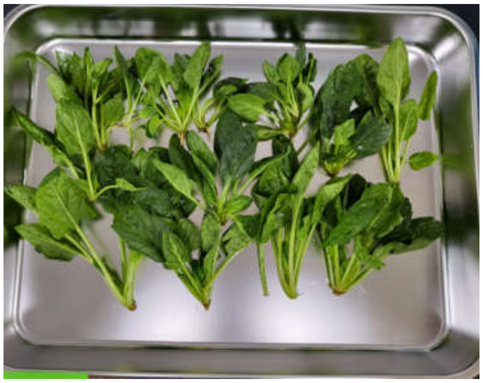
J

Ssamchoo

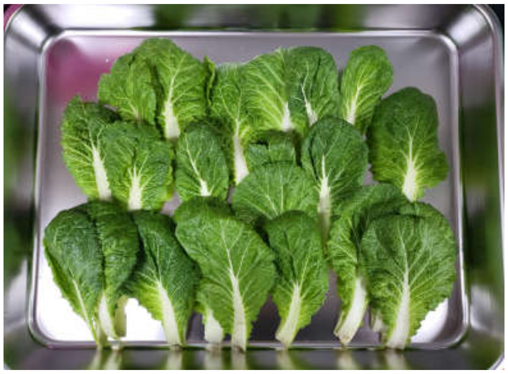
A

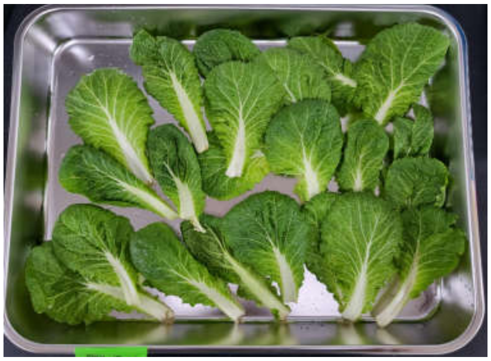
B

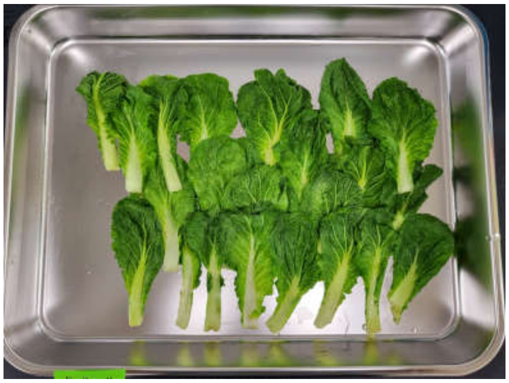
C

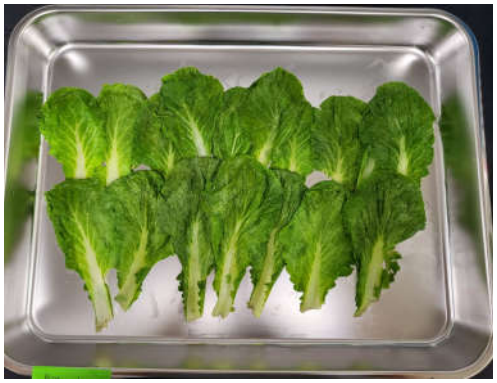
D

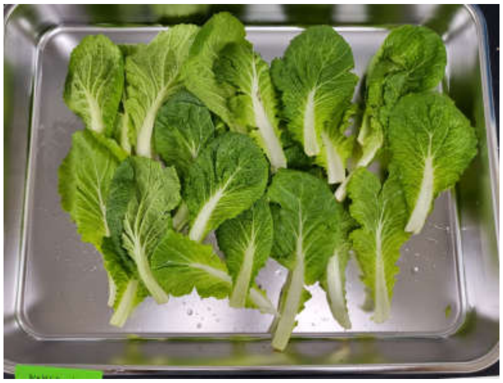
E

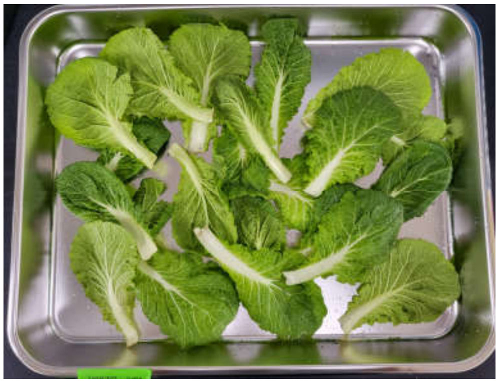
F

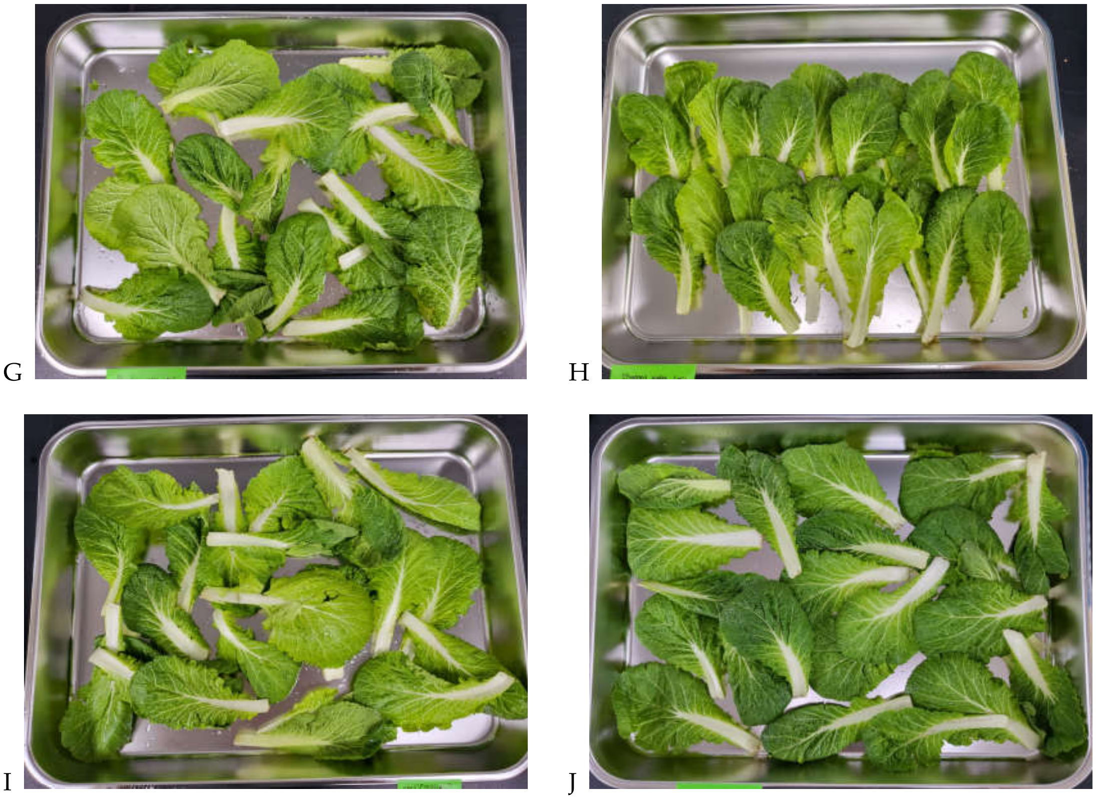

Figure S1. The effect of the treatment on the appearance of the vegetables (spinach and ssamchoo). A: Unwashed, B: Alkaline water washing, C: Blanching, D: Boiling, E: $NaHCO_3$, F: Detergent, G: Running water, H: Stagnant water, I: Ultrasonic cleaning, and J: Vinegar.
