# Supplementary material for: Effectiveness of Different Washing Strategies on Pesticide Residue Removal: The First Comparative Study on Leafy Vegetables
Source: Foods. 2022 Sep 19;11(18):2916. doi: 10.3390/foods11182916 (PMC9498324; doi:10.3390/foods11182916)
Supplement: Supplementary file 1 [file foods-11-02916-s001.zip › foods_supplementary_Table S1_Final .pdf]

Table S1. The results of nine washing methods for pesticide reduction in five leafy vegetables (n=5).

Pesticide	Treatment	Spinach		Ssamchoo		Crown daisy		Lettuce		Perilla leaves	
		Concentration (mg/kg)	% Reduction	Concentration (mg/kg)	% Reduction	Concentration (mg/kg)	% Reduction	Concentration (mg/kg)	% Reduction	Concentration (mg/kg)	% Reduction
Diniconazole	Initial	12.83 ± 0.46	-	10.97 ± 0.52	-	10.95 ± 0.89	-	15.56 ± 0.86	-	14.79 ± 0.35	-
	Blanching	7.71 ± 0.69	39.9	6.17 ± 0.35	43.8	9.38 ± 1.27	14.3	5.52 ± 0.12	64.6	8.32 ± 0.86	43.8
	Running water	1.81 ± 0.29	85.9	4.75 ± 1.03	56.7	2.93 ± 0.29	73.3	0.44 ± 0.00	97.1	2.69 ± 0.67	81.8
	Stagnant water	6.57 ± 0.76	48.8	6.22 ± 1.17	43.3	6.44 ± 0.87	41.1	5.78 ± 0.38	62.9	4.65 ± 0.76	68.6
	Boiling	5.31 ± 0.58	58.6	5.28 ± 1.07	51.9	4.92 ± 0.87	55.1	6.07 ± 0.69	61.0	12.07 ± 1.04	18.4
	Alkaline water	6.66 ± 0.68	48.1	5.91 ± 0.66	46.1	7.34 ± 1.17	32.9	3.49 ± 0.27	77.6	4.19 ± 0.27	71.7
	$NaHCO_3$	7.05 ± 0.75	45.1	5.61 ± 0.70	48.9	6.91 ± 0.62	36.9	3.79 ± 0.48	75.7	4.57 ± 0.43	69.1
	Vinegar	6.46 ± 1.06	49.7	6.12 ± 0.20	44.2	7.45 ± 0.59	31.9	4.22 ± 0.40	72.9	5.03 ± 0.72	66.0
	Detergent (CaO)	7.95 ± 1.09	38.0	7.00 ± 0.44	36.2	8.52 ± 1.15	22.2	6.25 ± 0.29	59.8	7.41 ± 0.61	49.9
	Ultrasonic cleaning	6.27 ± 0.14	51.1	7.50 ± 0.57	31.6	6.41 ± 0.60	41.5	3.97 ± 0.26	74.5	4.95 ± 0.09	66.5
Lufenuron	Initial	8.27 ± 0.34	-	4.88 ± 0.14	-	7.73 ± 0.49	-	8.07 ± 1.04	-	9.04 ± 0.21	-
	Blanching	6.43 ± 0.48	22.3	4.66 ± 0.29	4.6	4.41 ± 0.36	42.9	3.98 ± 0.32	50.6	6.92 ± 0.76	23.5
	Running water	1.83 ± 0.37	77.9	2.63 ± 0.42	46.2	2.06 ± 0.28	73.4	1.58 ± 0.12	80.5	1.89 ± 0.29	79.1
	Stagnant water	4.15 ± 0.54	49.8	4.01 ± 0.47	17.9	3.57 ± 0.34	53.9	3.48 ± 0.27	56.9	3.89 ± 0.37	56.9
	Boiling	5.28 ± 0.71	36.1	5.99 ± 0.77	-22.6	5.05 ± 0.21	34.6	5.58 ± 1.32	30.9	9.49 ± 0.69	-5.0
	Alkaline water	4.86 ± 0.50	41.2	3.78 ± 0.56	22.6	3.21 ± 0.19	58.5	2.71 ± 0.15	66.4	3.52 ± 0.13	61.0
	$NaHCO_3$	4.71 ± 0.65	43.0	3.83 ± 0.41	21.6	2.81 ± 0.11	63.7	3.12 ± 0.24	61.3	3.60 ± 0.13	60.2
	Vinegar	4.48 ± 0.91	45.8	3.73 ± 0.21	23.6	3.68 ± 0.46	52.4	3.08 ± 0.32	61.8	4.06 ± 0.09	55.1
	Detergent (CaO)	4.36 ± 0.55	47.3	2.96 ± 0.13	39.3	3.97 ± 0.29	48.7	2.89 ± 0.19	64.2	4.12 ± 0.55	54.4

	Ultrasonic cleaning	4.90 ± 0.27	40.8	4.45 ± 0.59	8.9	3.67 ± 0.30	52.5	3.08 ± 0.08	61.9	3.51 ± 0.30	61.1
Azoxystrobin	Initial	37.04 ± 1.52	-	27.96 ± 0.91	-	29.67 ± 1.40	-	34.81 ± 3.13	-	42.97 ± 0.59	-
	Blanching	11.94 ± 1.21	67.8	8.36 ± 0.70	70.1	6.01 ± 0.40	79.7	6.50 ± 0.61	81.3	13.63 ± 1.06	68.3
	Running water	1.35 ± 0.38	96.4	3.87 ± 0.44	86.2	2.99 ± 0.25	89.9	2.74 ± 0.23	92.1	3.68 ± 0.88	91.4
	Stagnant water	18.23 ± 1.55	50.8	13.65 ± 1.46	51.2	11.33 ± 0.40	61.8	11.81 ± 1.15	66.1	11.13 ± 2.27	74.1
	Boiling	5.57 ± 0.70	85.0	5.11 ± 1.24	81.7	2.31 ± 0.21	92.2	4.42 ± 1.13	87.3	14.66 ± 1.22	65.9
	Alkaline water	17.90 ± 1.61	51.7	13.41 ± 1.84	52.0	10.87 ± 0.66	63.4	7.85 ± 0.55	77.5	9.26 ± 0.53	78.5
	$NaHCO_3$	19.41 ± 1.68	47.6	16.67 ± 1.80	40.4	9.80 ± 0.32	67.0	9.99 ± 1.08	71.3	12.51 ± 1.02	70.9
	Vinegar	22.09 ± 2.70	40.4	16.18 ± 1.05	42.1	12.42 ± 1.31	58.1	9.57 ± 1.36	72.5	14.54 ± 0.68	66.2
	Detergent (CaO)	16.11 ± 0.49	56.5	16.32 ± 1.01	41.6	18.46 ± 0.90	37.8	15.08 ± 0.60	56.7	22.27 ± 2.21	48.2
	Ultrasonic cleaning	17.68 ± 2.69	52.3	16.21 ± 1.94	42.0	11.06 ± 0.70	62.7	8.93 ± 0.17	74.4	13.64 ± 0.28	68.3
Indoxacarb	Initial	16.32 ± 0.76	-	10.86 ± 1.09	-	16.65 ± 1.04	-	8.72 ± 1.44	-	20.76 ± 2.72	-
	Blanching	10.69 ± 1.06	34.5	5.99 ± 0.36	44.8	16.52 ± 2.67	0.8	3.85 ± 0.48	55.8	9.37 ± 0.59	54.9
	Running water	1.95 ± 0.29	88.1	4.46 ± 1.03	58.9	2.93 ± 0.29	82.4	0.21 ± 0.01	97.6	1.85 ± 0.42	91.1
	Stagnant water	7.53 ± 0.73	53.8	9.81 ± 1.61	9.6	6.44 ± 0.87	61.3	2.88 ± 0.53	67.0	3.28 ± 0.68	84.2
	Boiling	7.82 ± 1.07	52.1	4.78 ± 0.78	56.0	10.52 ± 1.90	36.8	2.73 ± 0.66	68.7	12.61 ± 1.76	39.3
	Alkaline water	6.64 ± 0.47	59.3	2.10 ± 0.39	80.7	8.73 ± 2.26	47.6	1.35 ± 0.13	84.5	1.98 ± 0.64	90.5
	$NaHCO_3$	7.00 ± 0.76	57.1	7.01 ± 1.26	35.4	8.33 ± 1.63	50.0	1.40 ± 0.15	84.0	4.56 ± 0.72	78.0
	Vinegar	5.46 ± 0.86	66.5	8.15 ± 0.88	24.9	5.82 ± 0.98	65.1	1.86 ± 0.21	78.7	3.44 ± 0.97	83.4
	Detergent (CaO)	7.91 ± 1.44	51.5	7.46 ± 0.32	31.3	10.96 ± 1.38	34.2	2.04 ± 0.14	76.6	7.67 ± 0.70	63.0
	Ultrasonic cleaning	6.69 ± 0.28	59.0	1.95 ± 0.28	82.0	6.41 ± 0.60	61.5	1.55 ± 0.39	82.2	2.59 ± 0.43	87.5
Chlorfenapyr	Initial	13.49 ± 0.62	-	11.77 ± 0.53	-	11.54 ± 0.89	-	16.27 ± 0.72	-	15.44 ± 0.63	-
	Blanching	10.65 ± 0.81	21.0	8.34 ± 0.43	29.1	13.60 ± 2.19	-17.9	8.23 ± 0.43	49.4	9.08 ± 1.08	41.2

	Running water	2.06 ± 0.39	84.8	5.16 ± 0.58	56.1	2.49 ± 0.28	78.4	0.49 ± 0.01	97.0	2.55 ± 0.65	83.5
	Stagnant water	7.90 ± 0.77	41.4	6.67 ± 0.98	43.3	7.78 ± 0.97	32.5	6.65 ± 0.70	59.1	4.69 ± 0.74	69.6
	Boiling	8.56 ± 1.16	36.5	9.49 ± 1.27	19.3	9.89 ± 1.58	14.3	12.90 ± 1.34	20.7	15.33 ± 1.02	0.7
	Alkaline water	7.25 ± 0.67	46.2	6.38 ± 0.78	45.8	8.15 ± 1.64	29.4	4.36 ± 0.22	73.2	3.67 ± 0.29	76.2
	$NaHCO_3$	7.75 ± 0.87	42.6	7.54 ± 0.55	36.0	7.60 ± 0.70	34.1	5.16 ± 0.42	68.3	4.93 ± 0.54	68.1
	Vinegar	6.89 ± 1.17	48.9	6.22 ± 0.60	47.1	8.55 ± 0.49	25.9	5.52 ± 0.57	66.1	5.71 ± 0.85	63.0
	Detergent (CaO)	9.33 ± 1.28	30.9	6.98 ± 0.53	40.7	9.23 ± 1.27	20.0	7.59 ± 0.48	53.3	8.25 ± 0.92	46.6
	Ultrasonic cleaning	7.88 ± 0.20	41.5	7.94 ± 0.55	32.6	7.38 ± 0.67	36.0	4.45 ± 0.47	72.6	5.47 ± 0.15	64.6
	Initial	30.38 ± 1.52	-	23.12 ± 0.72	-	21.64 ± 1.21	-	34.00 ± 4.21	-	39.28 ± 0.97	-
	Blanching	16.71 ± 1.68	45.0	11.49 ± 0.92	50.3	10.13 ± 1.00	53.2	10.93 ± 1.07	67.8	19.48 ± 1.93	50.4
	Running water	4.14 ± 0.88	86.4	6.13 ± 0.77	73.5	8.56 ± 0.78	60.4	4.31 ± 0.32	87.3	6.24 ± 1.22	84.1
	Stagnant water	17.58 ± 1.58	42.1	13.07 ± 1.65	43.5	13.83 ± 1.42	36.1	12.73 ± 1.33	62.5	14.01 ± 2.62	64.3
Fludioxonil	Boiling	8.84 ± 1.06	70.9	8.99 ± 1.99	61.1	4.64 ± 0.42	78.6	9.78 ± 2.37	71.2	22.91 ± 2.06	41.7
	Alkaline water	16.86 ± 2.00	44.5	13.06 ± 1.87	43.5	13.80 ± 1.08	36.2	8.42 ± 0.64	75.2	12.01 ± 0.44	69.4
	$NaHCO_3$	19.21 ± 1.62	36.7	16.57 ± 2.55	28.3	11.75 ± 0.72	45.7	9.87 ± 1.23	71.0	14.60 ± 0.84	62.8
	Vinegar	17.23 ± 2.26	43.3	15.56 ± 0.90	32.7	15.06 ± 2.21	30.4	9.79 ± 1.24	71.2	17.29 ± 1.79	56.0
	Detergent (CaO)	20.74 ± 3.54	31.7	14.83 ± 1.03	35.8	15.68 ± 1.26	27.5	14.91 ± 0.59	56.2	26.13 ± 1.96	33.5
	Ultrasonic cleaning	15.73 ± 0.71	48.2	16.27 ± 2.05	29.6	14.21 ± 1.18	34.3	9.71 ± 0.29	71.4	15.77 ± 0.10	59.9
	Initial	4.52 ± 0.24	-	2.95 ± 0.12	-	4.02 ± 0.27	-	4.45 ± 0.60	-	5.74 ± 0.12	-
	Blanching	1.35 ± 0.28	70.1	0.85 ± 0.07	71.3	0.65 ± 0.05	83.9	0.56 ± 0.08	87.3	1.83 ± 0.14	68.1
Chlorantraniliprole	Running water	0.09 ± 0.07	98.1	0.37 ± 0.07	87.3	0.20 ± 0.03	95.1	0.39 ± 0.04	91.2	0.86 ± 0.07	84.9
	Stagnant water	2.51 ± 0.23	44.4	1.67 ± 0.24	43.5	1.40 ± 0.04	65.3	1.55 ± 0.18	65.1	1.76 ± 0.19	69.3
	Boiling	0.66 ± 0.10	85.4	0.44 ± 0.15	85.1	0.17 ± 0.03	95.7	0.34 ± 0.13	92.4	1.84 ± 0.16	67.9

	Alkaline water	2.50 ± 0.24	44.6	1.64 ± 0.28	44.4	1.32 ± 0.11	67.1	0.94 ± 0.10	79.0	1.50 ± 0.05	73.8
	$NaHCO_3$	3.10 ± 0.26	31.4	2.39 ± 0.44	18.8	1.19 ± 0.05	70.4	1.39 ± 0.15	68.7	1.85 ± 0.07	67.9
	Vinegar	2.72 ± 0.38	39.8	2.24 ± 0.25	24.0	1.64 ± 0.26	59.2	1.27 ± 0.21	71.6	2.21 ± 0.12	61.4
	Detergent (CaO)	3.22 ± 0.66	28.8	2.02 ± 0.14	31.6	2.42 ± 0.20	39.9	2.23 ± 0.12	49.8	3.30 ± 0.40	42.5
	Ultrasonic cleaning	2.49 ± 0.11	44.8	2.02 ± 0.28	31.5	1.29 ± 0.08	67.8	1.09 ± 0.03	75.5	2.05 ± 0.03	64.2
Imidacloprid	Initial	11.62 ± 0.59	-	8.54 ± 0.40	-	13.13 ± 1.28	-	12.31 ± 1.54	-	16.03 ± 0.54	-
	Blanching	2.17 ± 0.24	81.4	1.31 ± 0.15	84.6	1.67 ± 0.36	87.3	1.42 ± 0.24	88.5	2.19 ± 0.31	86.4
	Running water	1.91 ± 0.26	83.6	5.61 ± 0.27	34.3	2.65 ± 0.29	79.8	6.56 ± 0.55	46.8	8.97 ± 1.54	44.0
	Stagnant water	5.35 ± 0.45	54.0	7.02 ± 1.01	17.8	6.70 ± 0.52	49.0	7.18 ± 0.39	41.7	10.50 ± 1.50	34.5
	Boiling	0.64 ± 0.06	94.5	0.67 ± 0.10	92.1	0.38 ± 0.06	97.1	0.87 ± 0.14	92.9	1.50 ± 0.12	90.7
	Alkaline water	5.31 ± 0.45	54.3	6.98 ± 1.01	18.3	6.32 ± 0.31	51.9	5.56 ± 0.62	54.9	9.01 ± 0.38	43.8
	$NaHCO_3$	5.27 ± 0.84	54.6	8.24 ± 1.15	3.5	5.59 ± 0.29	57.5	6.63 ± 0.51	46.1	8.69 ± 0.64	45.8
	Vinegar	4.74 ± 0.79	59.2	7.66 ± 1.01	10.3	5.91 ± 0.68	55.0	6.89 ± 0.48	44.1	10.23 ± 1.90	36.2
	Detergent (CaO)	5.71 ± 1.13	50.9	7.20 ± 0.51	15.7	6.58 ± 0.56	49.9	6.54 ± 0.56	46.9	10.94 ± 1.27	31.8
	Ultrasonic cleaning	6.01 ± 0.24	48.3	7.78 ± 0.76	9.0	5.43 ± 0.30	58.6	6.69 ± 0.11	45.6	9.82 ± 0.23	38.7
Thiamethoxam	Initial	13.24 ± 0.68	-	9.27 ± 0.48	-	17.34 ± 1.96	-	15.46 ± 1.96	-	17.35 ± 0.56	-
	Blanching	2.94 ± 0.30	77.8	1.67 ± 0.18	82.0	2.56 ± 0.68	85.2	1.93 ± 0.43	87.5	2.53 ± 0.44	85.4
	Running water	1.96 ± 0.28	85.2	6.36 ± 0.37	31.4	3.25 ± 0.43	81.3	7.85 ± 0.63	49.2	10.59 ± 2.13	39.0
	Stagnant water	5.76 ± 0.47	56.5	7.57 ± 1.12	18.3	8.32 ± 0.78	52.0	8.73 ± 0.60	43.5	10.84 ± 1.58	37.5
	Boiling	0.79 ± 0.11	94.0	0.74 ± 0.11	92.0	0.39 ± 0.12	97.8	0.99 ± 0.16	93.6	1.36 ± 0.13	92.2
	Alkaline water	5.77 ± 0.57	56.4	7.42 ± 1.05	20.0	7.68 ± 0.32	55.7	6.80 ± 0.82	56.0	10.73 ± 0.46	38.2
	$NaHCO_3$	5.82 ± 0.89	56.0	9.25 ± 1.26	0.2	6.79 ± 0.41	60.8	8.34 ± 0.70	46.0	10.46 ± 0.85	39.7
	Vinegar	5.15 ± 0.91	61.1	8.48 ± 1.26	8.6	7.06 ± 0.77	59.3	8.37 ± 0.59	45.8	12.21 ± 2.10	29.6

	Detergent (CaO)	6.04 ± 1.31	54.4	8.05 ± 0.61	13.2	7.32 ± 0.61	57.8	7.92 ± 0.73	48.7	12.87 ± 1.46	25.8
	Ultrasonic cleaning	6.58 ± 0.24	50.3	8.29 ± 0.79	10.6	6.56 ± 0.33	62.2	7.88 ± 0.18	49.0	9.74 ± 0.26	43.9
Pyraclostrobin	Initial	22.04 ± 0.87	-	16.65 ± 0.29	-	19.20 ± 0.91	-	22.35 ± 2.73	-	26.95 ± 0.65	-
	Blanching	14.78 ± 2.25	32.9	9.63 ± 1.10	42.2	9.72 ± 0.88	49.4	10.86 ± 1.08	51.4	14.67 ± 0.98	45.6
	Running water	1.86 ± 0.41	91.5	5.56 ± 0.98	66.6	5.53 ± 0.69	71.2	3.14 ± 0.31	85.9	4.33 ± 1.20	83.9
	Stagnant water	9.15 ± 1.17	58.5	8.53 ± 1.04	48.7	8.37 ± 0.92	56.4	7.68 ± 0.33	65.6	7.61 ± 1.07	71.7
	Boiling	12.63 ± 4.35	42.7	10.50 ± 1.66	36.9	8.30 ± 0.47	56.8	12.13 ± 2.79	45.7	19.33 ± 1.06	28.3
	Alkaline water	9.62 ± 0.95	56.4	8.92 ± 1.80	46.4	8.30 ± 0.57	56.8	5.44 ± 0.36	75.7	6.72 ± 0.28	75.1
	$NaHCO_3$	9.47 ± 1.20	57.0	8.82 ± 0.73	47.0	7.22 ± 0.41	62.4	5.76 ± 0.66	74.2	7.40 ± 0.36	72.6
	Vinegar	8.09 ± 1.48	63.3	8.10 ± 0.43	51.4	8.91 ± 1.15	53.6	6.08 ± 0.69	72.8	8.23 ± 1.23	69.5
	Detergent (CaO)	11.14 ± 1.89	49.4	9.84 ± 0.66	40.9	9.97 ± 0.91	48.1	8.41 ± 0.64	62.4	10.05 ± 0.85	62.7
	Ultrasonic cleaning	8.66 ± 0.49	60.7	11.02 ± 1.31	33.8	9.26 ± 0.68	51.8	6.14 ± 0.10	72.5	9.01 ± 0.14	66.6
